# Antioxidant Activities of Co-Encapsulated Natal Plum (*Carissa macrocarpa*) Juice Inoculated with *Ltp. plantarum 75* in Different Biopolymeric Matrices after In Vitro Digestion

**DOI:** 10.3390/foods11142116

**Published:** 2022-07-16

**Authors:** Faith Seke, Vimbainashe E. Manhivi, Marie Guerin, Tinotenda Shoko, Stephen A. Akinola, Cyrielle Garcia, Fabienne Remize, Retha M. Slabbert, Dharini Sivakumar

**Affiliations:** 1 Department of Horticulture, Tshwane University of Technology, Pretoria 0001, South Africa; fayeesk@gmail.com (F.S.); SlabbertMM@tut.ac.za (R.M.S.); 2 Phytochemical Food Network Group, Department of Crop Sciences, Tshwane University of Technology, Pretoria 0001, South Africa; ManhiviVE@tut.ac.za (V.E.M.); shokot@tut.ac.za (T.S.); akinolasa@tut.ac.za (S.A.A.); 3 Qualisud, Univ Montpellier, Univ de La Réunion, CIRAD, Institut Agro, Avignon Université, F-34398 Montpellier, France; marie.guerin@univ-reunion.fr (M.G.); cyrielle.garcia@univ-reunion.fr (C.G.); Fabienne.remize@univ-reunion.fr (F.R.); 4 SupAgro et Univerité de Montpellier, INRAE, Insitut Agro Montpellier, F-34000 Montpellier, France

**Keywords:** indigenous fruit, anthocyanins, lactic acid bacteria, biopolymers, antioxidant power and scavenging activity, simulated gastrointestinal digestion

## Abstract

Biopolymeric systems that co-encapsulate probiotics and bioactive compounds ensure timely delivery in the gastrointestinal tract. Cyanidin 3-sambubioside is the dominant anthocyanin in Natal plum (*Carissa macrocarpa*). This study aims at the co-encapsulation of Natal plum (*Carissa macrocarpa*) juice inoculated with *Lactiplantibacillus plantarum* 75 (*Ltp. plantarum 75*) by freeze-drying using pea protein isolate, maltodextrin, and psyllium mucilage and evaluating their release in vitro. An encapsulation efficiency of >85% was noted in lactic acid bacteria (LAB) survival and anthocyanin content. Freeze-drying produced pinkish-red powder, rich in polyphenols and LAB (>6 Log CFU mL^−1^) after 14 days of storage. Natal plum juice + maltodextrin + pea protein isolate + psyllium mucilage + *Ltp. plantarum 75* (NMPeaPsyB) showed the highest LAB population (6.74 Log CFU mL^−1^) with a survival rate of 81.9%. After digestion, NMPeaPsyB and NMPeaPsy had the highest LAB survival (>50%) at 67.5% and 67.5 ± 0.75%, respectively, and the highest bioaccessibility of cyanidin 3-sambubioside in Natal plum juice than the other co-encapsulation with other biopolymers. NMPeaPsy and NMPeaPsyB showed phenolic stability in the gastric phase and controlled release in the intestinal simulated phase. The antioxidant activities had strong correlations with cyanidin 3-sambubioside. The results confirmed that microencapsulation is important for improving stability and allowing for the development of functional foods.

## 1. Introduction

Natal plum (*Carissa macrocarpa*) is a traditional fruit from southern Africa that has become popular due to its polyphenol content. Cyanidin 3-O-sambubioside, cyanidin 3-O-pyranoside, and cyanidin 3-O-glucoside were identified as the major polyphenols in Natal plum fruit extracts [1,2]. Cyanidin derivatives have been linked to various health benefits such as anti-influenza effects [3] and anti-diabetic properties [4]. The antioxidant properties of Natal plum are linked to cyanidin derivatives and other phenolic acids such as coumaric acid, syringic acid, dicaffeoyl tartaric acid, protocatechuic acid, caffeic acid, chlorogenic acid, ellagic acid, ferulic acid, and catechin [2]. The benefits of polyphenols have been found to be dependent not only on their intake but also on their bioaccessibility [5]. In our previous study, the bioaccessibility of cyanidin 3-sambubioside (C-3-S) in Natal plum extract was reported to be 32.2% [2]. It is therefore critical to increase polyphenol bioaccessibility so that a significant amount of polyphenols reaches the colon, where they are depolymerized into simpler forms and improve body health.

Therefore, to improve the consumers’ health, it is crucial to introduce the plant’s bioactive compounds and probiotics into their diet. Furthermore, probiotics contribute to improved gastrointestinal health [6]. Combining bioactive compounds and probiotics, through food or supplements, is beneficial for enhancing their nutritional impact [6]. However, incorporating probiotics into foods presents certain concerns, as ingestion of probiotic bacteria-containing foods is ineffective without proper protection against adverse effects [6]. The bioaccessibility of probiotics is affected by high-temperature and high-acidity conditions [7]. Polyphenols and probiotics both require protection, and microencapsulation is one of the most effective ways to encapsulate sensitive chemicals in a coating or wall material. The wall material protects the sensitive compounds from unfavorable responses and controls their release in the gastrointestinal tract [7]. In the encapsulation process, selection of wall materials is important. Various wall materials (maltodextrin, guar gum, gum Arabic, and proteins) have been used to encapsulate polyphenols and probiotic bacteria [7]. Under common storage and gastrointestinal settings, the combination of materials with diverse qualities confers superior protective features and appropriate release characteristics [8]. 

Maltodextrins are polymers of saccharides that consist of glucose units, primarily linked by α-1,4 glucosidic bonds. Maltodextrin has provided excellent protection to anthocyanin in encapsulated blackcurrant [9] and purple sweet potato [10]. Maltodextrin protects encapsulated food ingredients from oxidation and boosts their stability [11]. Gandia-Herrero et al. [12] successfully used 20% of maltodextrin to encapsulate the bioactive pigment of opuntia fruits. However, it has been reported that maltodextrin has a high glycemic index value of 185–105 and should be consumed sparingly by diabetic and/or hypoglycemic individuals [13]; hence, it could be appropriate to use lower maltodextrin levels in meals and add other low glycemic index matrices during food processing. According to Fang and Bhandari [14], a single encapsulating matrix does not contain all of the needed features; hence, efforts to improve encapsulation properties have been made by combining proteins and polysaccharides. Polymer blends that potentially result in higher encapsulating efficiency and lower cost than individual biopolymers have piqued researcher curiosity.

The combination of diverse biopolymeric matrices also improves the morphological and mechanical qualities, which theoretically can be manipulated to produce food with customized properties [7]. Proteins such as pea protein isolates are also an intriguing wall material because of their low cost and food-grade variety. Psyllium mucilage is a plant-based arabinoxylan biopolymer and can boost the growth of probiotic bacteria in the gut and help with a variety of digestive issues [15]. Psyllium has also been suggested in combination with alginate for co-encapsulating probiotic bacteria, resulting in products with higher encapsulation yields and greater stability than other prebiotics [16]. Furthermore, psyllium enhances the stability of encapsulated components when the pH is lowered, which makes it useful in acidic products such as yogurt [17]. Therefore, it seems promising to co-encapsulate Natal plum juice inoculated with *Lactiplantibacillus plantarum* 75 using a combination of maltodextrin, pea protein and/or psyllium mucilage to improve phenolic bioaccessibility and lactic acid bacteria viability after storage and gastrointestinal digestion.

The purpose of this study was to study the effect of different compositions of biopolymers on the encapsulation efficiency, phenolic and anthocyanin content, and antioxidant activity of Natal plum juice inoculated with *Ltp. plantarum 75* as well as to characterize and quantify the phenolics present in Natal plum juice and to determine the effects of encapsulating materials on cell viability and phenolic bioaccessibility when exposed to in vitro gastrointestinal conditions.

## 2. Materials and Methods

### 2.1. Plant Material and Reagents

Natal plum (Carissa Macrocarpa) fruits were harvested at the red stage of maturity between December 2020 and January 2021 (summer) from the Tshwane University of Technology, Pretoria campus (25°43′55.6′′ S, 28°09′ 52.3′′ E) gardens, Pretoria, South Africa. After harvest, fruits were washed using tap water and deseeded and stored at −80 °C until further analysis. 

All chemicals were purchased from Sigma Aldrich Co. (Johannesburg, Gauteng, South Africa). The chemical reagents, standards, and solvents used were of analytical grade. 

### 2.2. Preparation of Culture

*Ltp plantarum* 75 (L75) was obtained from the culture collections held by the University of La Réunion and QualiSud, France. It was isolated from cabbage. *Ltp. plantarum 75* cells were activated by adding 2 g of *Ltp. plantarum 75* capsules to 100 mL of sterile MRS broth and incubating at 37 °C for 24 h, following Phromthep and Leenanon [18]. The *Ltp. plantarum 75* cultures were then centrifuged (HermLe Z326k, HermLe Labortechnik, Wehingen, Germany) at 5000× *g* at 4 °C for 10 min. The pellets were harvested and centrifuged with phosphate buffer saline solution and thereafter suspended into 50 mL of phosphate buffer saline solution.

### 2.3. Development of Natal Plum Juice

**Figure 1:**
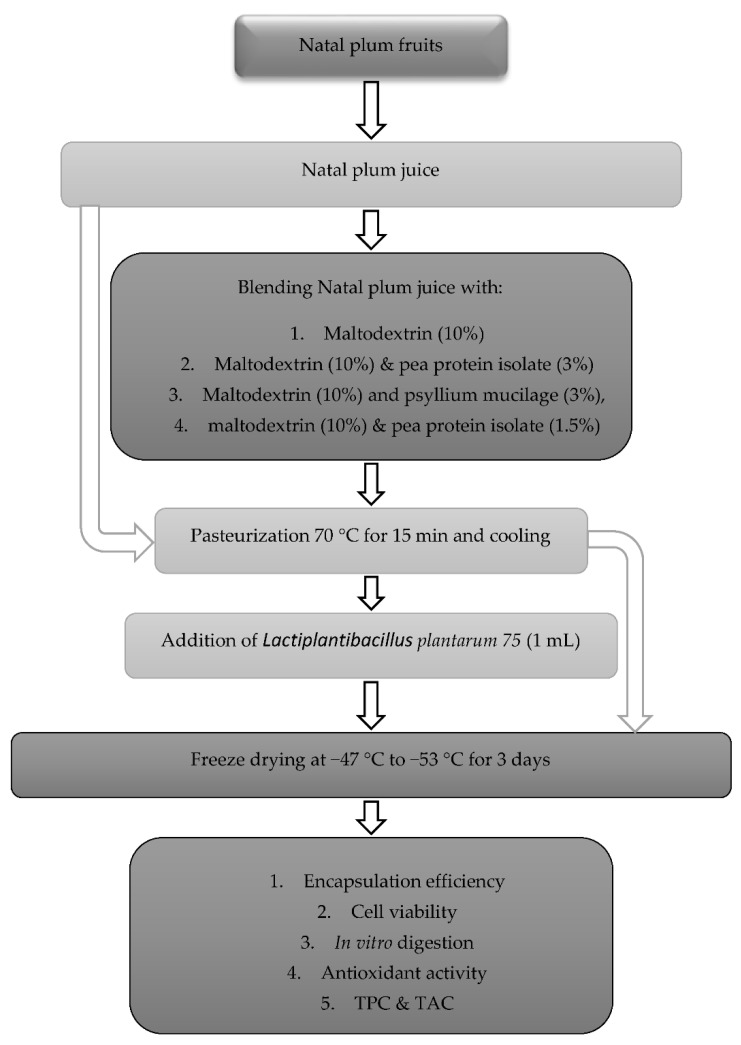
Schematic representation of the experimental design.

The development of the co-encapsulated Natal plum juice inoculated with *Ltp. plantarum 75* in different biopolymerics is illustrated in Figure 1 and was developed following a method described by Vanajakshi et al. [19] with some modifications. The Natal plum (100 g) fruit was homogenized using a blender, and 200 mL of water was added. Then, the mixture was homogenized again for 2 min. Ten formulations were obtained by blending the Natal plum juice with maltodextrin, maltodextrin and pea protein isolate, maltodextrin, pea protein isolate and psyllium mucilage and maltodextrin and psyllium mucilage with or without lactic acid bacteria (Table 1). The juice samples were then pasteurized at 70 °C in a water bath for 15 min. Each juice sample contained 200 mL pasteurized Natal plum juice, 200 mL of biopolymer dissolved in water, and 1 mL of lactic bacteria in an isotonic solution. The control juices did not have any biopolymers. The resulting Natal plum juice was then freeze-dried after 48 h of freezing using a Benchtop Freeze Dryer (VirTis Sp Scientific, Model # 2kBTES-55, Gardiner, NY, USA) at −47 to −53 °C. The freeze-dried powders were stored in brown bottles with screw caps and stored at 4 °C.

### 2.4. In Vitro Digestion of Natal Plum (Carissa macrocarpa) Juice Extract

To test the in vitro digestibility profile and stability of polyphenols and *Ltp. plantarum 75* survival in co-microencapsulated powder. The co-microencapsulated powder samples were exposed to simulated gastric juice at pH 2.0 and intestinal juice at pH 7.7, as described by Chen et al. [20]. In addition, an enzyme-containing digestion blank without the material was stored at −80 °C. According to Equation (1), we calculated the percentage release of the Ltp plantarum 75 and then the bioaccessibility (what was in the dialysis tube) using the following:

(1) $$\mathrm{Bioaccessibility}\%=(\frac{B_{SI}\;\;}{B_{ND}})\times 100$$

*B_SI_* (mg kg^−1^) is the phenolic compound content in the dialysis tube, and *B_ND_* (mg kg^−1^) is the phenolic compound content in the undigested sample.

### 2.5. Extraction of Phenolic Compounds in Natal Plum (Carissa macrocarpa) Juice Powder

Freeze-dried extracts of micro-encapsulated Natal plum juice biopolymer inoculated with or without *Ltp. plantarum 75* was prepared using a method described by Seke et al. [2], with slight modifications. Each sample (100 mg) was extracted using 38% ethanol (10 mL) and ultrasonicated for 30 min at 40 °C and then centrifuged (Hermle Z326k, Hermle Labortechnik GmbH, Wehingen, Germany) at 1000× *g* for 30 min at 4 °C. The extracts were subjected to HPLC-DAD analysis for total phenolic content (TPC), total anthocyanin content (TAC), antioxidant capability, and different phenolic compounds.

### 2.6. Total Phenolic Content (TPC)

TPC of samples was determined using modified Folin–Ciocalteu method [1]. Encapsulated and un-encapsulated Natal plum juice extract (10 µL) was mixed with 100 µL of a 10% Folin–Ciocalteu reagent and 1 mL distilled water. After 8 min, 300 µL of 10% NaHCO3 was added. The solutions were left to stand for 60 min in a dark room. The blank sample was also prepared, containing 10 µL methanol, 100 µL of 10% Folin–Ciocalteu reagent and 300 µL of 10% NaHCO_3_. The absorbance was measured at 765 nm. The TPC was quantified using the gallic acid (GA) as standard, and the calibration curve was constructed. The phenolic content in the micro-encapsulated Natal plum juice extract containing biopolymers inoculated with or without *Ltp. plantarum 75* was expressed in terms of the gallic acid equivalent mg GAE kg^−1^ DW. 

### 2.7. Total Anthocyanin Content (TAC)

TAC was determined using the pH differential method described by Mendes et al. [21]. Encapsulated and un-encapsulated Natal plum juice extracts (10 µL) were diluted with 920 µL of 0.025 mol.L^−1^ KCl pH 1.0 and 0.4 mol.L^−1^ CH_3_CO_2_Na pH 4.5 buffers to achieve the same dilution. The absorbance was measured at 510 and 700 nm in both pH = 1.0 and pH = 4.5 buffers, and the results were expressed as mg Cyanidin 3-glucoside (Cyd-3-glu) 100^−1^ g dry weight of powder.

The encapsulation efficiency was calculated using Equation (2), as described by Mendes et al. [21], as follows.

(2) $$\mathrm{EE}\%=\frac{Anthocyanin\;content\;in\;microbeads}{Anthocyanin\;content\;in\;the\;extract}\;\times \;100$$

The lactic acid bacteria encapsulation efficiency was determined using a method described by Colín-Cruz et al. [8] and Maciel et al. [22]. The quantification of the viable bacteria was conducted by the pour plate method. The percentage (%) of the encapsulation efficiency was calculated using Equation (3).

(3) $$\mathrm{EE}\%=\frac{N}{N_{0}}\;\times \;100$$

where *N* is the number of viable cells (CFU g^−1^) in the powder, and *N_o_* is the number of viable cells in the solution before the freeze-drying.

### 2.8. Viability of Lactic Acid Bacteria

To determine the viability of *Ltp. plantarum 75* cells, the unencapsulated and encapsulated Natal plum juice powder was redissolved in sterile water. A 12-fold serial dilution was performed by using sterile peptone water and the pour plate method was adopted based on Güney and Güngörmüşler [23]. The cells were cultivated on the MRS-agar plates after 48 h of aerobic incubation at 37 °C to estimate the number of colony-forming units (CFU). The counts were expressed as CFU mL^−1^ DW.

### 2.9. Quantification of Targeted Phenolic Metabolites

Natal plum juice samples with the highest survival of *Ltp. plantarum 75* after storage and after in vitro digestion were selected for phenolic acid characterization quantification, and the unencapsulated Natal plum juice samples were used as controls. The phenolic compounds of interest (anthocyanins and phenolic acids) were quantified by High-Performance Liquid Chromatography with Diode-Array Detection (HPLC-DAD) Model Flexar TM 89173-556 (PerkinElmer, Waltham, Massachusetts, USA), as described by Seke et al. [2]. Extracts from the different Natal plum juices (with and without lactic acid bacteria, before and after digestion) were filtered with PTFE membrane filters, followed by phenolic running on a Waters HSS T3 C18, 2.1100 mm, 1.7 mm column. Pure external standards were used for detection and quantification. Supplementary Table S1 provides the regression equations. Chromatograms were constructed at wavelengths 320 and 520 nm (phenolic acids, flavonoids, and anthocyanins), and the chromatograms are shown in Supplementary Figures S1–S4.

### 2.10. Determination of the Antioxidant Activity of Phenolic Extracts

The antioxidant activity of the Natal plum juice samples was determined using three different assays, namely 2,2-diphenyl-1-picrylhydrazyl (DPPH) [1], and 2,2-azino-bis (3-ethylbenzothiazoline-6-sulfonic acid) (ABTS) radical scavenging assay [2] and the Ferric Reducing Antioxidant Potential (FRAP) assay following a method described by Seke et al. [24]. The Natal plum juice samples with the best *Ltp. plantarum 75* survival during storage and after in vitro digestion were selected for antioxidant activity assays compared to the unencapsulated Natal plum juice samples. DPPH was measured by taking the absorbance measurement of the extracts at 515 nm, and the results were represented as the antioxidant concentration required to reduce DPPH absorbance by 50% (IC_50_). The decrease in absorbance at 734 nm at a concentration of 40 µL of the sample (ABTS+) was measured using a multiplate reader (EZ Read 2000; Biocrom Ltd., Cambridge, UK) to calculate the IC_50_. The antioxidant capacity (FRAP assay) was expressed as Trolox-equivalent antioxidant capacity (mM TEAC g^−1^).

### 2.11. Statistical Analysis

Data in this study were expressed as mean ± standard deviation of at least three independent replicates. The significant differences were determined at *p* ˂ 0.05 using an analysis of variance (ANOVA) and Tukey’s multiple range test. The nonlinear regression was used to calculate dose–response inhibition of DPPH, ABTS and FRAP scavenging activities. A Pearson’s correlation analysis was performed on phenolic content, individual phenolic compounds, and antioxidant activities. Minitab Statistical Software for Windows (Minitab Statistical Software 20.3, Coventry, UK) was used to analyze the data.

## 3. Results and Discussion

### 3.1. Encapsulation Efficiency of Ltp. plantarum 75 and Anthocyanin in Natal Plum (Carissa macrocarpa) Juices-Biopolymer Powder

**Figure 2:**
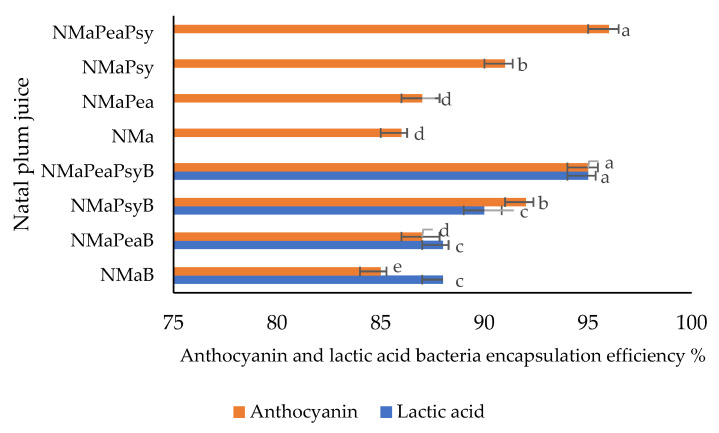
Lactic acid bacteria and anthocyanin encapsulation efficiency of co-encapsulated Natal plum (*Carissa macrocarpa*) juice. Error bars show the standard deviation value; bars with different letter are significantly different *p* < 0.05. Key: NMa: Natal plum (*Carissa macrocarpa*) juice + maltodextrin; NMaB: Natal plum (*Carissa macrocarpa*) juice + maltodextrin + *Ltp. plantarum 75;* NMaPea: Natal plum (*Carissa macrocarpa*) juice + maltodextrin + pea protein isolate; NMaPeaB: Natal plum (*Carissa macrocarpa*) juice + maltodextrin + pea protein isolate + *Ltp. plantarum 75;* NMaPsy: Natal plum (*Carissa macrocarpa*) juice + maltodextrin + psyllium mucilage; NMaPsyB: Natal plum (*Carissa macrocarpa*) juice + maltodextrin + psyllium mucilage + *Ltp. plantarum 75*; NMaPeaPsy: Natal plum (*Carissa macrocarpa*) juice + maltodextrin + pea protein isolate + psyllium mucilage; NMaPeaPsyB: Natal plum (*Carissa macrocarpa*) juice + maltodextrin + pea protein isolate + psyllium mucilage + *Ltp. plantarum 75*.

The encapsulation process allowed for a dark pink powder to be formed, with the encapsulation efficiency of anthocyanin ranging between 85% to 96%. The lowest encapsulation efficiency (EE) of 85% was observed for the Natal plum juice sample with maltodextrin (NMa). The addition of pea protein and psyllium mucilage to the Natal plum juice resulted in an increase in efficiency to 96% (Figure 2). The structures of the biopolymers added to the juice samples might have contributed to the higher efficiency of NmaPeaPsy (96%) and NmaPeaPsyB (95%). Covalently linked protein molecules to the carbohydrate chain act as good film-formers, which improve the encapsulation of the molecule and increase the stability of anthocyanins by making the flavylium cation less vulnerable to nucleophilic attack by water molecules [25]. A further possibility is that the complex nature created by the anthocyanins’ flavylium cation in interaction with dextrin prevented the anthocyanins from transforming into a less stable form. Similar encapsulation efficiency ranges of 78.61% and 92.98% have been reported for chokeberry fruit extracts encapsulated in maltodextrin, guar gum, gum Arabic, pectin, beta-glucan and inulin [26]. 

The lactic acid bacteria encapsulation efficiency of different encapsulated Natal plum juice samples is shown in Figure 2. The highest encapsulation efficiency (EE%) was observed for the Natal plum juice sample with a combination of maltodextrin, pea protein isolate and psyllium mucilage (NmaPeaPeaB) 95%. Natal plum juice containing maltodextrin (NmaB) had the lowest content (88%). Similar results were published by Colín-Cruz et al. [7], and the encapsulation efficiency of lactic acid bacteria was found to be 93.3% in whey protein concentrate and 89.4% in gum Arabic. Similar results have been reported by Colín-Cruz et al. [7], showing the encapsulation efficiency of lactic acid bacteria to be 93.3% in whey protein concentrate and 89.4% in gum Arabic.

Moreover, Bosnea, et al. [27] microencapsulated two different bacterial strains of *Lactobacillus paracasei* and *Lactobacillus paraplantarum* using complicated coacervation with whey protein isolate and gum Arabic and reported an encapsulation efficiency greater than 94.3%. Based on these findings, the combined use of maltodextrin, pea protein isolate, and psyllium mucilage as wall material could help deliver high-percent preservation and release of *Ltp. plantarum 75*. 

### 3.2. Effect of Storage Time on the Survival of Ltp. plantarum 75 in Natal Plum (Carissa macrocarpa) Juices

Table 2 shows the surviving population of *Ltp. plantarum 75* encapsulated and non-encapsulated Natal plum juice biopolymer matrices upon storage. Storage duration and encapsulation had a significant effect on the survival of *Ltp. plantarum 75* (*p* < 0.05). After 14 days of storage, the final survival counts for NMB, NMaB, NMaPeaB, NMaPsyB, and NMaPeaPeaB samples were 3.48, 4.08, 4.32, 5.33 and 6.74 log CFU mL^−1^, respectively. In all encapsulated samples, survival was higher than 50% until 14 days of storage than the control (NMB 48%). The low sugar content in NM with the biopolymer could preserve the LAB. The present findings indicate that a synergistic effect is operating, whereby maltodextrin protects the pea protein and psyllium mucilage microencapsulants from fragmentation during storage while the pea protein and psyllium mucilage matrix protect the bacteria from membrane damage by maltodextrin. Ding and Shah [28] compared the survival rates of free and microencapsulated bacteria in orange and apple juices, demonstrating that encapsulation effectively protects against the acidic nature of these juices. Studies have shown that psyllium coating improves the viability of *Lb. acidophilus* during storage [29]. Accordingly, microencapsulation provides a suitable environment and a physical barrier to probiotic bacteria [28]. The findings of the present study also reviewed that after 14 days of storage, NMaPeaPeaB had a bacteria population > 1 × 10^6^ CFU mL^−1^, which was above the standard concentration expected for LABs in the gut, which could suggest its functions as a probiotic. Since in this study no fermentation was performed, we assume that polyphenols had no direct effect on the survival of lactic acid bacteria. These results demonstrate that the bacterial culture used survived in the Natal plum juice supplemented with different biopolymers, with a greater survival rate for the NMaPeaPsyB sample.

### 3.3. Effect of In Vitro Digestion on the Survival of Ltp. plantarum 75 from Natal Plum Juices

**Figure 3:**
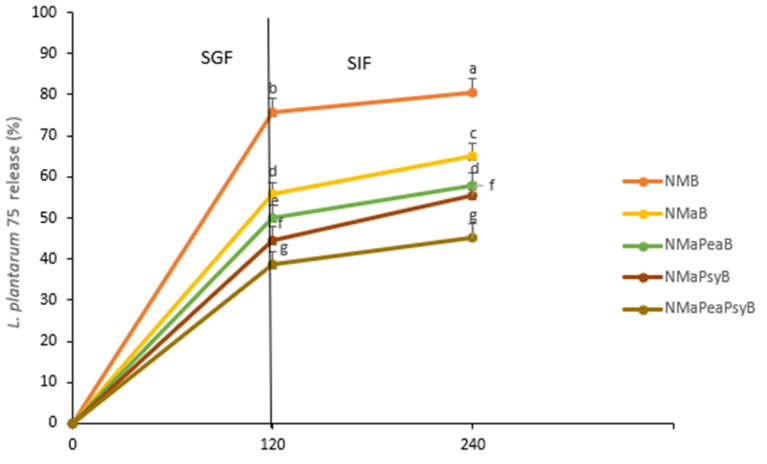
Release of *Ltp. plantarum 75* in simulated gastric fluid (SGF) for 120 min, sequentially added to the simulated intestinal fluid (SIF) until complete 240 min of the assay, expressed as percentages (%), considering the initial number of viable cells in Natal plum (*Carissa macrocarpa*) juice. Error bars show the standard deviation value; bars with different letters are significantly different *p* < 0.05. Key: NMaB: Natal plum (*Carissa macrocarpa*) juice + maltodextrin + *Ltp. plantarum 75*; NMaPeaB: Natal plum (*Carissa macrocarpa*) juice + maltodextrin + pea protein isolate + *Ltp. plantarum 75*; NMaPsyB: Natal plum (*Carissa macrocarpa*) juice + maltodextrin + psyllium mucilage + *Ltp. plantarum 75*; NMaPeaPsyB: Natal plum (*Carissa macrocarpa*) juice + maltodextrin + pea protein isolate + psyllium mucilage + *Ltp. plantarum 75*.

The different Natal plum juice samples inoculated with *Ltp. plantarum 75* were subjected to simulated gastrointestinal digestion, and the release rate was evaluated (Figure 3). The highest rate of release of *Ltp plantarum 75* from NMB during the gastric digestion phase (SGF) and intestinal digestion phase (SIF) was 5.76% and 80.65%, respectively.

NMaPeaPsyB, which was the best performing sample, released less than 50% of *Ltp. plantarum 75* at the intestinal digestion. Co-encapsulation allowed for *Ltp. plantarum 75* to be bioaccessible after intestinal digestion. The polysaccharides in the maltodextrin–pea protein isolate–psyllium matrix might have formed conjugates that prevented some soluble polysaccharides from dissociating in the GI tract, thus protecting *Ltp. plantarum 75*.

Huang et al. [30] reported that the buffering capacity of protein isolates such as pea protein isolate, and polysaccharides protect probiotic microorganisms from digestive stress. It is also possible that the physical–chemical characteristics of Natal plum juices delayed the release of probiotics, possibly because bioactive compounds interact with the polymers that form the capsules. To fully experience prebiotic and probiotic effects, the bacteria and polyphenols need to be protected so that they reach the colony where they will influence each other [31]. Our results indicated that co-encapsulation of prebiotics and probiotics with biopolymers enhanced the survival of *Ltp. plantarum 75* during gastrointestinal digestion.

### 3.4. Effect of In Vitro Digestion on the Total Phenolic Content (TPC) and Total Anthocyanin Content (TAC) from Natal Plum (Carissa macrocarpa) Juice

Table 3 shows the TPC and TAC of Natal plum juice-biopolymer extract. The highest TPC (859.52 ± 3.76 mg GAE kg^−1^ DW) was observed in NMaPeaPsy. The lowest TPC was found in NMB (836.53 ± 2.98 mg GAE kg^−1^ DW) and NM (838.38 ± 4.89) with no significant difference between them. The highest TAC was observed for NMaPeaPsy (54.32 ± 0.56 mg Cy-Glu 100^−1^ g DW) and NMaPeaPsyB (53.23 ± 0.68 mg Cy-Glu 100^−1^ g DW) while the least was observed for NM (40.27 ± 0.27 mg Cy-Glu 100^−1^ g DW) and NMB (40.03 ± 0.79 mg Cy-Glu 100^−1^ g DW). 

After exposure to gastrointestinal conditions, both TPC and TAC reduced in all the samples. Natal plum encapsulated with different biopolymers showed bioaccessibility of TPC ranging from 37% to 55%. Of all biopolymer encapsulations with Natal plum, NM showed the least bioaccessible percentage (37%), while NMaPeaPsy had the highest of 55%. The bioaccessibility of TAC increased from 43% (NM) to 62% (NMaPeaPsy) after intestinal digestion. Therefore, co-encapsulation improved the bioaccessibility of TAC after exposure to gastrointestinal digestion. The findings of this study are consistent with those of Flores et al. [32], who investigated the in vitro release of encapsulated blueberry extracts and found a higher bioaccessibility of total phenol content (68%) after intestinal digestion. Saikia et al. [33] also found that the release of phenolic compounds from *Averrhoa carambola* pomace microencapsulated with maltodextrin by spray and freeze-drying was higher in the gastric-simulated medium (pH 2.0) than in simulated intestinal medium in both procedures (pH 7.7).

The microencapsulate behavior in simulated gastrointestinal media is influenced by the type and properties of the encapsulating covering material, as well as their tolerance or resistance to digestive enzymes and gastrointestinal circumstances such as pH range [34]. Polysaccharides such as psyllium mucilage have been shown to have numerous hydroxyl groups (OH-) in their chemical structure, which improves the hydrophilic aspect of the molecule. The presence of hydroxyl groups in Natal plum juice biopolymer samples probably allowed for the creation of hydrogen bonds with water. In this way, hydroxyl groups help to produce the coating matrix by promoting contact between the wall material and the encapsulated compounds while also protecting the encapsulated substance [7]. Our results showed that encapsulated phenolic compounds are protected from the effect of changes in pH during digestion. The results of this study showed the significant potential of using a combination of maltodextrin, pea protein isolate and psyllium mucilage to encapsulate polyphenols and anthocyanins, allowing for targeted release.

### 3.5. In Vitro Digestibility of Natal Plum (Carissa macrocarpa) Juice Polyphenols

The cyanidin 3-O-sambubioside (C-3-S) was the predominant anthocyanin in nondigested encapsulated and unencapsulated Natal plum juice extract. It reached the highest content (484 ± 4.23 mg kg^−1^) with the encapsulated NMaPeaPsy while the least amount of content (464.5 ± 2.79 mg kg^−1^) was found in the unencapsulated (NMB) (Table 4). Our previous study [24] showed that the bioaccessibility of a C-3-S from the Natal plum extract was 32.2%, and the encapsulation process improved the bioaccessibility of C-3-S in this study. The increase in protocatechuic acid and ferulic acid in the intestinal fraction corresponded with the breakdown of C-3-S, resulting in an increase in their bioaccessibility.

Ferulic acid and protocatechuic acid are byproducts of the degradation of cyanidin derivatives [24]. Thus, encapsulating the Natal plum juice resulted in improved phenolic acids bioaccessibility when compared to our previous study [2]. The bioaccessibility of phenolics during digestion depends on their stability when exposed to different pH, the structure of the compound, fruit matrix, solubility, and degradation, glycosylation, or esterification with other compounds [34]. According to Ortega et al. [35], the composition of the food matrix may influence the behavior of phenolic acids during digestion. The hydrolysis of gallotannins and ellagitannins could explain the increased amounts of ellagic acids after intestinal digestion [36]. pH 7 and bile salts may contribute to the loss of other phenolic acids in the intestinal phase, including chlorogenic acid, caffeic acid, and syringic acid [37]. Dietary polyphenols have been shown to aid in the maintenance of human health, particularly in the gut, by stimulating the growth of beneficial bacteria while inhibiting the growth of pathogenic bacteria, resulting in a prebiotic-like effect [38]. 

The majority of the ingested polyphenols do not reach the colon because polyphenols are poorly absorbed or metabolized by human enzymes during their passage through the upper gastrointestinal tract. Therefore, the results of this study showed that the encapsulation process improved the number of polyphenols that survived in the gastrointestinal digestion conditions and made them bioaccessible in the colon.

### 3.6. Effect of In Vitro Gastrointestinal Digestion on the Antioxidant Activities of Natal Plum (Carissa macrocarpa) Juice Phenolic Extract

**Figure 4:**
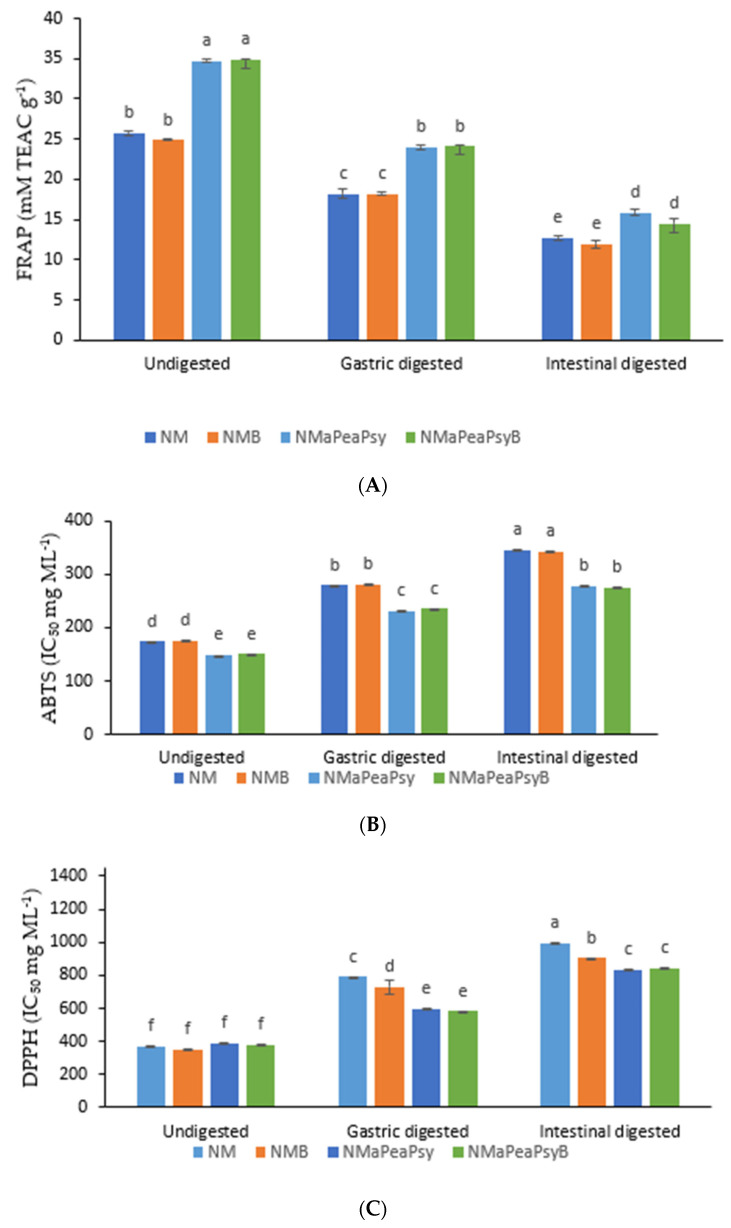
Effect of co-encapsulation of Natal plum (*Carissa macrocarpa*) juice on FRAP (**A**), ABTS (**B**) and DPPH (**C**) scavenging activity. Error bars show the standard deviation value, bars with different letters are significantly different *p* < 0.05. Key: NM: Natal plum (*Carissa macrocarpa*) juice; NMB: Natal plum (*Carissa macrocarpa*) juice + *Ltp. plantarum 75*; NMaPsy: Natal plum (*Carissa macrocarpa*) juice + maltodextrin + psyllium mucilage; NMaPsyB: Natal plum (*Carissa macrocarpa*) juice + maltodextrin + psyllium mucilage + *Ltp. plantarum 75*; NMaPeaPsy: Natal plum (*Carissa macrocarpa*) juice + maltodextrin + pea protein isolate + psyllium mucilage; NMaPeaPsyB: Natal plum (*Carissa macrocarpa*) juice + maltodextrin + pea protein isolate + psyllium mucilage + *Ltp. plantarum 75*.

**Figure 5:**
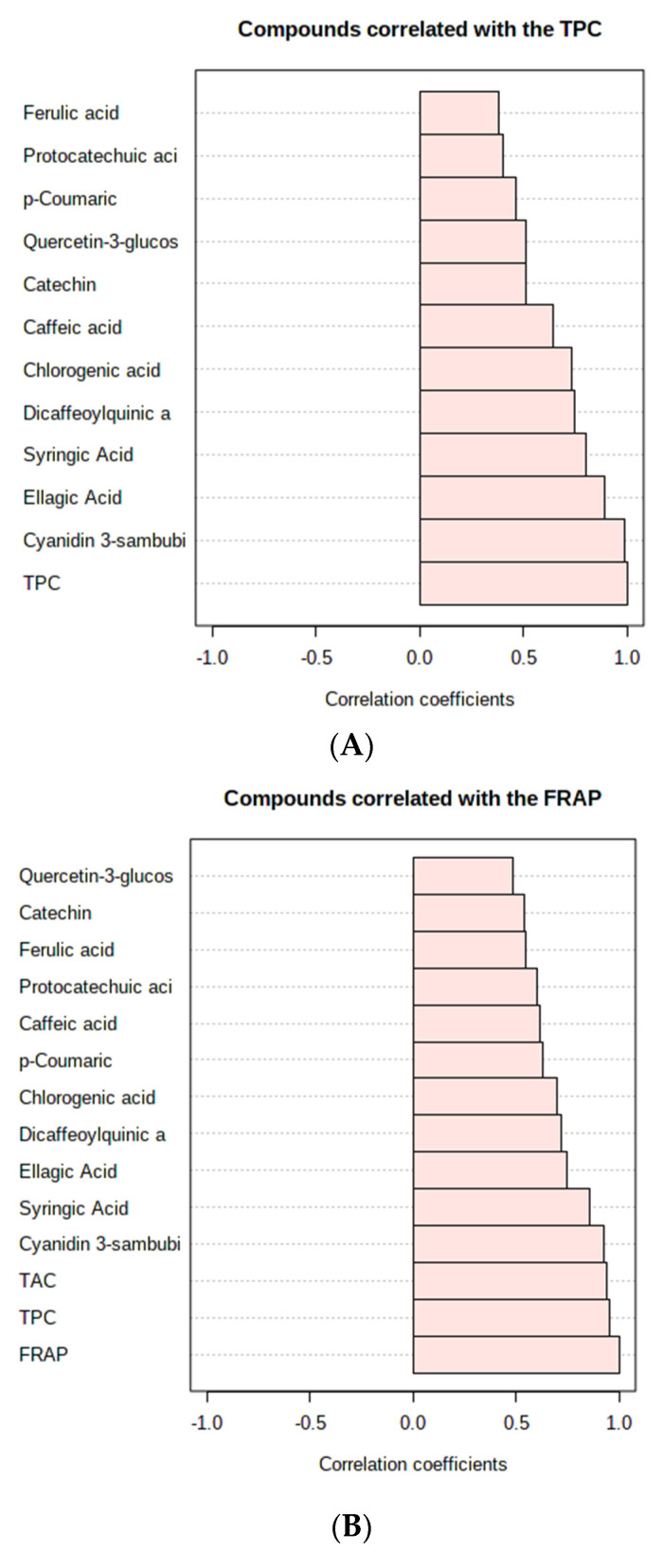

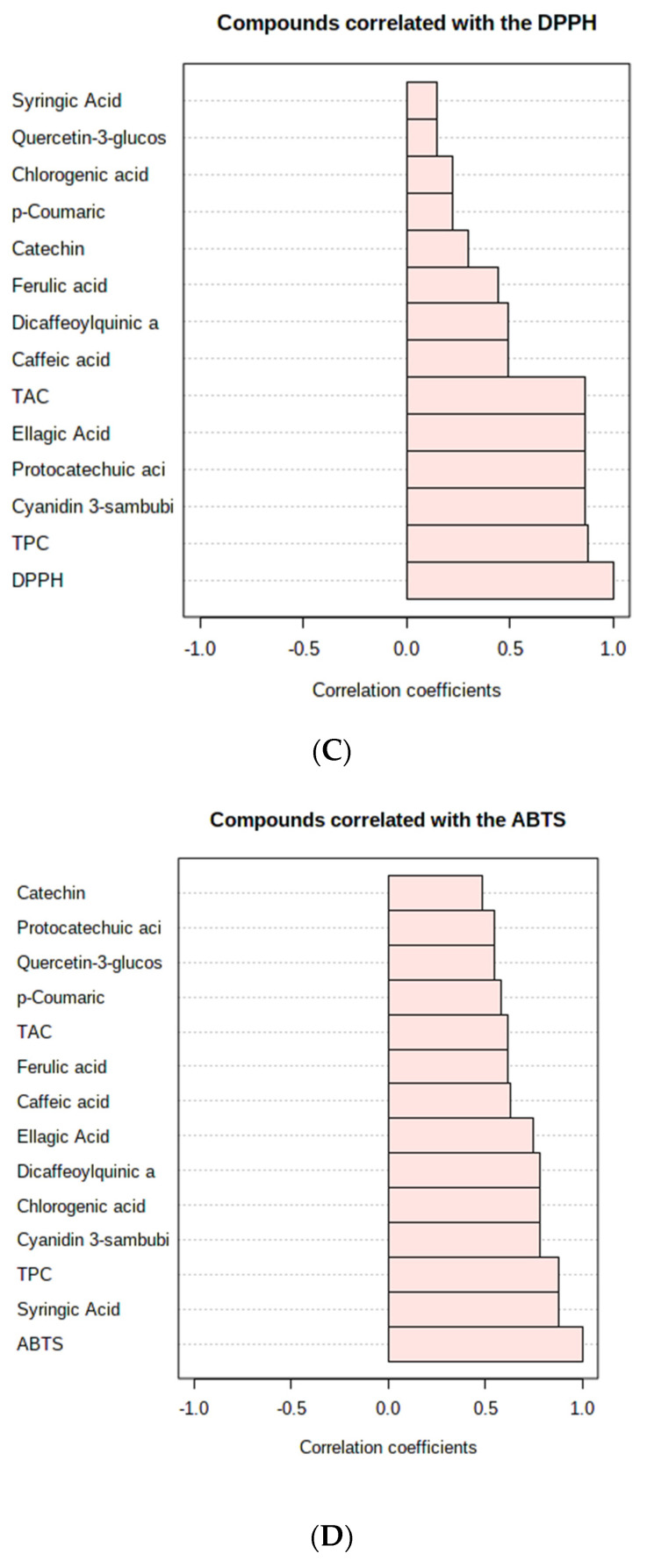
Correlation of total phenolic content (**A**), FRAP antioxidant capacity (**B**), DPPH (**C**) and ABTS (**D**) radical scavenging capacities with phenolic compounds.

Figure 4A–C shows the antioxidant activities of encapsulated and unencapsulated Natal plum juice extracts inoculated with or without *Ltp. plantarum 75*. Antioxidant activities (FRAP, ABTS and DPPH) decreased during digestion, and this could be attributed to phenolics being depleted after digestion. However, the encapsulated Natal plum juice possessed superior antioxidant activities compared to unencapsulated Natal plum juice. This can be attributed to the protective properties provided by the encapsulating wall material used. In addition, because of the potential of wall materials to donate electrons or hydrogen, they can participate in antioxidant processes, increasing the antioxidant activity of encapsulated food components [39]. The antioxidant properties of plant extracts are usually attributed to the presence of phenolic components. However, the changes that occur during gastrointestinal digestion can alter the chemical structure of the phenolic components, resulting in varying antioxidant capabilities [40]. A strong positive correlation exists between TPC and FRAP (*r* = 0.95), TAC and FRAP (*r* = 0.94), Cy-3-Sa (*r* = 0.93), syringic acid (0.85), and ellagic acid (0.74), which was significant at *p* < 0.01 (Figure 5).

Similarly, a strong positive correlation between FRAP and TPC, TPC and anthocyanins has been observed previously by Seke et al. [2] and Manhivi et al. [41]. The antioxidant power (FRAP) also showed a positive correlation with all phenolic compounds identified in the Natal plum juice. The ABTS radical scavenging showed a strong positive correlation with TPC (0.87), ellagic acid (0.75), syringic acid (0.86), Cy-3-Sa (0.80) and chlorogenic acid (0.78). The DPPH radical scavenging activity showed a positive correlation with all the identified phenolic compounds, TPC and TAC. TPC (0.94), Cy-3-Sa (0.86), protocatechuic acid (0.86), ellagic acid (0.85), and TAC were found to have the strongest correlations with DPPH (0.87). Thus, these findings imply that phenolic components played an important role in the antioxidant activity of encapsulated Natal plum juice. Phenolics’ antioxidant action is largely determined by their molecular structure. The CHCHCOOH grouping of hydroxycinnamic acids has been found to have a greater capacity to transfer a proton and to stabilize radicals later than the carboxyl (COOH) grouping of hydroxybenzoic acids [42]. Furthermore, flavonoids alone, depending on their structure, might operate as protons or electron donors, resulting in a strong association. The difference in activity could be attributable to the compounds’ synergistic or antagonistic effects.

## 4. Conclusions

The bioaccessibility of cyanidin 3-sambubioside after intestinal digestion was highest in NMPeaPsy and NMPeaPsyB. The degradation of cyanidin 3-sambubioside correlated with the increase in protocatechuic acid and ferulic acid in the intestinal phase. A strong correlation was observed between cyanidin 3-sambubioside, protocatechuic acid, ellagic acid, syringic acid, chlorogenic acid, TPC and TAC against antioxidant (DPPH, ABTS, FRAP) activities. Moreover, the incorporation of maltodextrin, pea protein isolates and psyllium mucilage are promising since it provided greater protection to *Ltp. plantarum 75,* anthocyanins and phenolic compounds in vitro. Data suggest that the Natal plum juice powder can be a natural and functional colorant in the food industry. However, more research on cellular models such as Caco-2 as well as animal models is required to acquire qualitative data for human investigations.

## Figures and Tables

**Table 1:** Sample formulations.

Sample	Code
Natal plum (*Carissa macrocarpa*) juice	NM
2. Natal plum (*Carissa macrocarpa*) juice + *Lactiplantibacillus plantarum 75*	NMB
3. Natal plum (*Carissa macrocarpa*) juice + maltodextrin	NMa
4. Natal plum (*Carissa macrocarpa*) juice + maltodextrin + *Ltp. plantarum 75*	NMaB
5. Natal plum (*Carissa macrocarpa*) juice + maltodextrin + pea protein isolate	NMaPea
6. Natal plum (*Carissa macrocarpa*) juice + maltodextrin + pea protein isolate + *Ltp. plantarum 75*	NMaPeaB
7. Natal plum (*Carissa macrocarpa*) juice + maltodextrin + psyllium mucilage	NMaPsy
8. Natal plum (*Carissa macrocarpa*) juice + maltodextrin + psyllium mucilage + *Ltp. plantarum 75*	NMaPsyB
9. Natal plum (*Carissa macrocarpa*) juice + maltodextrin + pea protein isolate + psyllium mucilage	NMaPeaPsy
10. Natal plum (*Carissa macrocarpa*) juice + maltodextrin + pea protein isolate + psyllium mucilage + *Ltp. plantarum 75*	NMaPeaPsyB

**Table 2:** Survival of the bacterial growth and survival percentage in Natal plum (*Carissa macrocarpa*) juices over a 14-day storage period.

Sample	Day 0 Log CFU mL^−1^	Day 7 Log CFU mL^−1^	Day 14 Log CFU mL^−1^
NMB	7.25 ^a^ ± 0.21	4.81 ^b^ ± 0.12 (66.34%)	3.48 ^c^ ± 0.78 (48%)
NMaB	7.29 ^a^ ± 0.46	5.37 ^b^ ± 0.82 (73.66%)	4.08 ^b^ ± 0.23 (55.96%)
NMaPeaB	7.79 ^a^ ± 0.3	5.78 ^b^ ± 0.73 (74.19%)	4.32 ^b^ ± 0.69 (55.45%)
NMaPsyB	7.85 ^a^ ± 0.83	6.12 ^b^ ± 0.36 (77.96%)	5.33 ^b^ ± 0.52 (67.89%)
NMaPeaPsyB	8.92 ^a^ ± 0.74	7.16 ^b^ ± 0.87 (80.3%)	6.74 ^b^ ± 0.58 (75.5%)

Means followed by the same letter ± standard deviation within the row is not significantly different at *p* < 0.05. The numbers in brackets indicate the survival of microorganisms in percentages. Key: NMB: Natal plum juice + *Ltp. plantarum 75;* NMaB: Natal plum (*Carissa macrocarpa*) juice + maltodextrin + *Ltp. plantarum 75*; NMaPeaB: Natal plum (*Carissa macrocarpa*) juice + maltodextrin + pea protein isolate + *Ltp. plantarum 75*; NMaPsyB: Natal plum (*Carissa macrocarpa*) juice + maltodextrin + psyllium mucilage + *Ltp. plantarum 75*; NMaPeaPsyB: Natal plum (*Carissa macrocarpa*) juice + maltodextrin + pea protein isolate + psyllium mucilage + *Ltp. plantarum 75*.

**Table 3:** An evaluation of the effect of simulated in vitro digestion on the total phenolic, total anthocyanin content of Natal plum (*Carissa macrocarpa*) juices.

Total Phenolic Content (mg GAE kg^−1^ DW)	Undigested	Gastric Digested	Intestinal Digested	Bioaccessibility (%)
NM	838.38 ^a^ ± 4.89	545.36 ^b^ ± 2.48	310.24 ^c^ ± 2.74	37.00 ^d^ ± 0.47
NMB	836.53 ^a^ ± 2.98	555.55 ^b^ ± 3.31	311.17 ^c^ ± 1.29	37.19 ^d^ ± 1.23
NMa	840.46 ^a^ ± 4.46	639.61 ^b^ ± 2.19	417.47 ^c^ ± 2.19	49.67 ^c^ ± 0.75
NMaB	840.83 ^a^ ± 2.27	635.82 ^b^ ± 3.13	416.73 ^c^ ± 1.25	49.56 ^c^ ± 0.92
NMaPea	847.19 ^a^ ± 5.92	675.01 ^b^ ± 1.28	428.18 ^c^ ± 3.93	50.00 ^c^ ± 0.28
NMaPeaB	845.69 ^a^ ± 4.37	671.96 ^b^ ± 3.79	429.09 ^c^ ± 2.20	52.60 ^b^ ± 0.74
NMaPsy	847.25 ^a^ ± 5.36	688.75 ^b^ ± 1.75	432.71 ^c^ ± 3.15	50.00 ^c^ ± 0.93
NMaPsyB	845.88 ^a^ ± 4.28	681.96 ^b^ ± 3.38	431.39 ^c^ ± 2.17	52.60 ^b^ ± 0.84
NMaPeaPsy	859.52 ^a ±^ 3.76	788.63 ^b^ ± 2.97	472.71 ^c^ ± 2.02	55.20 ^a^ ± 0.83
NMaPeaPsyB	858.73 ^a ±^ 3.82	781.06 ^b^ ± 1.79	473.39 ^c^ ± 1.67	55.00 ^a^ ± 0.96
Total anthocyanin content (mg Cy-Glu 100^−1^ g DW)				
NM	40.27 ^a^ ± 0.27	39.29 ^b^ ± 0.37	17.54 ^c^ ± 0.28	43.55 ^d^ ± 0.78
NMB	40.03 ^a^ ± 0.79	39.93 ^b^ ± 0.53	17.38 ^c^ ± 0.30	43.41 ^d^ ± 0.34
NMa	44.27 ^a^ ± 0.34	39.23 ^b^ ± 0.07	24.27 ^c^ ± 0.75	54.82 ^c^ ± 0.88
NMaB	44.03 ^a^ ± 0.53	39.46 ^b^ ± 0.18	23.88 ^c^ ± 0.83	54.23 ^c^ ± 0.34
NMaPea	45.98 ^a^ ± 0.82	36.49 ^b^ ± 0.35	25.29 ^c^ ± 0.71	55.00 ^c^ ± 0.64
NMaPeaB	45.79 ^a^ ± 0.64	36.38 ^b^ ± 0.72	25.09 ^c^ ± 0.45	54.79 ^c^ ± 0.73
NMaPsy	46.86 ^a^ ± 0.84	36.02 ^b^ ± 0.86	27.29 ^c^ ± 0.21	58.23 ^b^ ± 0.58
NMaPsyB	46.73 ^a^ ± 0.37	36.78 ^b^ ± 0.42	26.69 ^c^ ± 0.89	57.11 ^b^ ± 0.39
NMaPeaPsy	54.32 ^a^ ± 0.56	36.75 ^b^ ± 0.38	32.79 ^c^ ± 0.41	60.36 ^a^ ± 0.82
NMaPeaPsyB	53.23 ^a^ ± 0.68	36.61 ^b^ ± 0.65	33.01 ^c^ ± 0.23	62.00 ^a^ ± 0.71

Means followed by the same letter ± standard deviation within the row are not significantly different at *p* < 0.05. Means followed by the same letter ± standard deviation within the bioaccessibility column are not significantly different at *p* < 0.05. Key: NM: Natal plum (*Carissa macrocarpa*) juice; NMB: Natal plum (*Carissa macrocarpa*) juice + *Ltp. plantarum 75*; NMa: Natal plum (*Carissa macrocarpa*) juice + maltodextrin; NMaB: Natal plum (*Carissa macrocarpa*) juice + maltodextrin + *Ltp. plantarum 75*; NMaPea: Natal plum (*Carissa macrocarpa*) juice + maltodextrin + pea protein isolate; NMaPeaB: Natal plum (*Carissa macrocarpa*) juice + maltodextrin + pea protein isolate + *Ltp. plantarum 75*; NMaPsy: Natal plum (*Carissa macrocarpa*) juice + maltodextrin + psyllium mucilage; NMaPsyB: Natal plum (*Carissa macrocarpa*) juice + maltodextrin + psyllium mucilage + *Ltp. plantarum 75*; NMaPeaPsy: Natal plum (*Carissa macrocarpa*) juice + maltodextrin + pea protein isolate + psyllium mucilage; NMaPeaPsyB: Natal plum (*Carissa macrocarpa*) juice + maltodextrin + pea protein isolate + psyllium mucilage + *Ltp. plantarum 75*.

**Table 4:** Changes in phenolic components in Natal plum (*Carissa macrocarpa*) juice samples during gastrointestinal digestion for selected samples on a dry weight basis.

Phenolic Components (mg kg^−1^)	Undigested	Gastric	Intestinal	Bioaccessibility%
NM				
Cyanidin 3-sambubioside	467.00 ^a^ ± 3.87	329.40 ^b^ ± 1.00	196.54 ^c^ ± 2.71	42.00 ^l^ ± 0.58
Protocatechuic acid	32.30 ^a^ ± 1.03	16.01 ^c^ ± 0.63	19.92 ^b^ ± 0.87	61.50 ^f^ ± 0.62
Chlorogenic acid	47.99 ^a^ ± 0.29	22.27 ^b^ ± 0.29	15.38 ^c^ ± 0.83	32.10 ^m^ ± 0.23
Catechin	29.10 ^a^ ± 1.92	10.10 ^b^ ± 1.03	14.39 ^c^ ± 0.44	49.50 ^j^ ± 0.89
Quercetin-3-glucoside	53.37 ^a^ ± 0.21	38.19 ^b^ ± 0.82	24.01 ^c^ ± 0.39	44.00 ^k^ ± 0.49
Ferulic acid	27.99 ^a^ ± 0.79	15.09 ^c^ ± 0.22	19.28 ^b^ ± 0.87	68.80 ^b^ ± 0.75
Caffeic acid	25.75 ^a^ ± 1.28	19.05 ^b^ ± 0.68	12.65 ^c^ ± 0.79	49.10 ^h^ ± 0.49
p-Coumaric	28.07 ^a^ ± 0.49	12.40 ^b^ ± 0.65	17.28 ^c^ ± 0.18	61.60 ^f^ ± 0.28
Syringic Acid	15.40 ^a^ ± 4.27	7.23 ^b^ ± 1.80	9.47 ^c^ ± 1.03	61.50 ^f^ ± 0.68
Ellagic Acid	43.35 ^a^ ± 0.20	27.19 ^b^ ± 0.02	13.03 ^c^ ± 0.87	30.10 ^m^ ± 0.49
Dicaffeoylquinic acid	74.45 ^a^ ± 0.40	47.16 ^b^ ± 0.92	29.39 ^c^ ± 0.22	39.47 ^l^ ± 0.83
NMB				
Cyanidin 3-sambubioside	464.50 ^a^ ± 2.79	320.02 ^b^ ± 1.87	185.54 ^c^ ± 2.21	40.00 ^l^ ± 0.61
Protocatechuic acid	32.98 ^a^ ± 0.13	16.26 ^c^ ± 0.93	21.63 ^b^ ± 0.55	65.58 ^e^ ± 0.72
Chlorogenic acid	46.43 ^a^ ± 0.75	22.01 ^b^ ± 0.03	17.89 ^c^ ± 0.32	38.53 ^l^ ± 0.33
Quercetin-3-glucoside	54.27 ^a^ ± 0.38	38.39 ^b^ ± 0.21	22.01 ^c^ ± 0.19	40.50 ^l^ ± 0.58
Catechin	27.91 ^a^ ± 0.84	13.10 ^b^ ± 0.46	15.89 ^c^ ± 0.28	56.90 ^h^ ± 0.70
Ferulic acid	26.37 ^a^ ± 0.21	14.49.19 ^c^ ± 0.82	20.71 ^b^ ± 0.39	78.54 ^a^ ± 0.29
Caffeic acid	24.19 ^a^ ± 1.28	18.73 ^b^ ± 0.40	10.05 ^c^ ± 0.91	41.50 ^l^ ± 0.63
p-Coumaric acid	27.34 ^a^ ± 0.19	13.84 ^b^ ± 0.25	16.09 ^c^ ± 0.73	58.86 ^h^ ± 0.57
Syringic Acid	14.36 ^a^ ± 2.73	6.93 ^b^ ± 1.04	8.52 ^c^ ± 1.48	59.34 ^h^ ± 0.26
Ellagic Acid	42.73 ^a^ ± 0.72	25.22 ^b^ ± 0.23	12.74 ^c^ ± 0.59	29.80 ^m^ ± 0.38
Dicaffeoylquinic acid	72.28 ^a^ ± 0.39	47.23 ^b^ ± 0.62	27.09 ^c^ ± 0.53	37.47 ^l^ ± 0.49
NMaPeapsy				
Cyanidin 3-sambubioside	484.00 ^a^ ± 4.23	459.40 ^b^ ± 3.28	326.54 ^C^ ± 2.76	67.50 ^d^ ± 0.75
Protocatechuic acid	46.69 ^a^ ± 1.28	23.01 ^c^ ± 0.63	33.92 ^b^ ± 0.18	72.64 ^c^ ± 0.69
Chlorogenic acid	55.22 ^a^ ± 0.21	30.48 ^b^ ± 0.61	35.30 ^c^ ± 0.23	63.92 ^f^ ± 0.15
Catechin	32.89 ^a^ ± 1.03	16.10 ^b^ ± 0.92	20.31 ^c^ ± 0.68	61.70 ^f^ ± 0.49
Ferulic acid	28.75 ^a^ ± 0.13	19.09 ^c^ ± 0.41	24.28 ^b^ ± 0.87	84.40 ^a^ ± 0.51
Caffeic acid	46.47 ^a^ ± 1.70	35.04 ^b^ ± 0.83	27.81 ^c^ ± 0.92	59.80 ^g^ ± 0.27
Quercetin-3-glucoside	65.27 ^a^ ± 0.32	49.91 ^b^ ± 0.74	32.69 ^c^ ± 0.15	50.00 ^i^ ± 0.78
p-Coumaric	32.56 ^a^ ± 1.49	16.40 ^b^ ± 0.92	23.28 ^c^ ± 0.72	71.48 ^c^ ± 0.43
Syringic Acid	24.28 ^a^ ± 2.77	19.20 ^b^ ± 1.80	10.47 ^c^ ± 1.03	43.10 ^j^ ± 0.19
Ellagic Acid	53.35 ^a^ ± 1.20	38.19 ^b^ ± 0.92	28.74 ^c^ ± 0.81	53.87 ^h^ ± 0.95
Dicaffeoylquinic acid	72.34 ^a^ ± 1.44	42.84 ^b^ ± 1.59	39.09 ^c^ ± 0.76	54.00 ^h^ ± 0.70
NMaPeaPsyB				
Cyanidin 3-sambubioside	479.00 ^a^ ± 3.93	454.82 ^b^ ± 3.76	323.14 ^c^ ± 2.02	67.50 ^d^ ± 0.57
Protocatechuic acid	44.38 ^a^ ± 1.43	21.91 ^c^ ± 0.78	30.39 ^b^ ± 0.10	68.70 ^d^ ± 0.72
Chlorogenic acid	54.62 ^a^ ± 0.72	30.37 ^b^ ± 0.09	34.50 ^c^ ± 0.13	63.10 ^f^ ± 0.69
Quercetin-3-glucoside	64.87 ^a^ ± 0.45	49.03 ^b^ ± 0.32	36.01 ^c^ ± 0.90	55.50 ^h^ ± 0.44
Catechin	34.27 ^a^ ± 0.94	15.29 ^b^ ± 0.78	22.93 ^c^ ± 0.60	66.90 ^e^ ± 0.38
Ferulic acid	30.50 ^a^ ± 0.17	18.35 ^c^ ± 0.28	24.89 ^b^ ± 0.81	81.60 ^a^ ± 0.75
Caffeic acid	46.74 ^a^ ± 0.85	37.18 ^b^ ± 0.30	25.04 ^c^ ± 0.19	54.00 ^f^ ± 0.23
p-Coumaric	33.05 ^a^ ± 0.50	18.40 ^b^ ± 0.70	25.49 ^c^ ± 0.30	77.10 ^b^ ± 0.82
Syringic Acid	23.65 ^a^ ± 2.38	20.28 ^b^ ± 1.04	12.82 ^c^ ± 0.84	54.20 ^h^ ± 0.54
Ellagic Acid	52.95 ^a^ ± 1.74	36.90 ^b^ ± 0.59	30.04 ^c^ ± 0.62	57.00 ^h^ ± 0.93
Dicaffeoylquinic acid	74.94 ^a^ ± 1.12	42.04 ^b^ ± 0.75	37.59 ^c^ ± 0.28	50.10 ^i^ ± 0.47

Means followed by the same letter ± standard deviation within the row are not significantly different at *p* < 0.05. Means followed by the same letter ± standard deviation within the bioaccessibility column are not significantly different at *p* < 0.05. Key: NM: Natal plum juice; NMB: Natal plum juice + *Ltp. plantarum 75*; NMaPeaPsy: Natal plum juice + maltodextrin + pea protein isolate + psyllium mucilage; NMaPeaPsyB: Natal plum juice + maltodextrin + pea protein isolate + psyllium mucilage + *Ltp. plantarum 75*.

## Data Availability

Data available upon request.

## Supplementary Materials

The following supporting information can be downloaded at: https://www.mdpi.com/article/10.3390/foods11142116/s1. Figure S1: Chromatograms of Undigested Natal plum juice samples at 520 nm (anthocyanins); Figure S2: Chromatograms of Gastric digested Natal plum juice samples at 520 nm (anthocyanins): Figure S3: Chromatograms of Intestinal digested Natal plum juice samples at 520 nm (anthocyanins); Figure S4: Chromatograms of the identification of phenolic acids and flavonoids in Natal plum juice samples at 320 nm; Table S1: Retention time and regression equations for phenolic identification and quantification using HPLC-DAD.
