# Supplementary material for: Antioxidant Activities of Co-Encapsulated Natal Plum (Carissa macrocarpa) Juice Inoculated with Ltp. plantarum 75 in Different Biopolymeric Matrices after In Vitro Digestion
Source: Foods. 2022 Jul 16;11(14):2116. doi: 10.3390/foods11142116 (PMC9319165; doi:10.3390/foods11142116)
Supplement: Supplementary file 1 [file foods-11-02116-s001.zip › foods-1795282-supplementary.pdf]

Supplementary Table S1: Retention time and Regression equations for phenolic identification and quantification using HPLC-DAD

Phenolic	Retention time (min)	Regression equation	R^2	LOD (µg/L)	LOQ (µg/L)
Cyanidin 3-sambubioside	17. 924	y=14621x-3637	0.996	0.85	0.14
Protocatechuic acid	16.71	y=283211x+474.28	0.999	2.5	0.46
Chlorogenic acid	19.856	y=83920x-1552.2	0.995	0.27	0.37
Catechin	19.909	y=29372x-2482.6	0.994	2.39	0.79
Ferulic acid	29.412	y=29372x+902734	0.999	2.27	1.9
Caffeic acid	22.905	y=43642x-28362	0.999	1.86	3.2
p-Coumaric	28.07	y=201832x-22831	0.999	2.48	0.23
Syringic acid	23.525	y=72840x-2834.9	0.998	1.45	0.75
Ellagic acid	25.891	y=6932x+182932.73	0.995	1.31	1.2
Quercetin 3-glucoside	26.582	y=22783x+794.78	0.999	2.06	0.68
Dicaffeoylquinic acid	31.432	y=13407x-4418.9	0.995	1.36	0.87

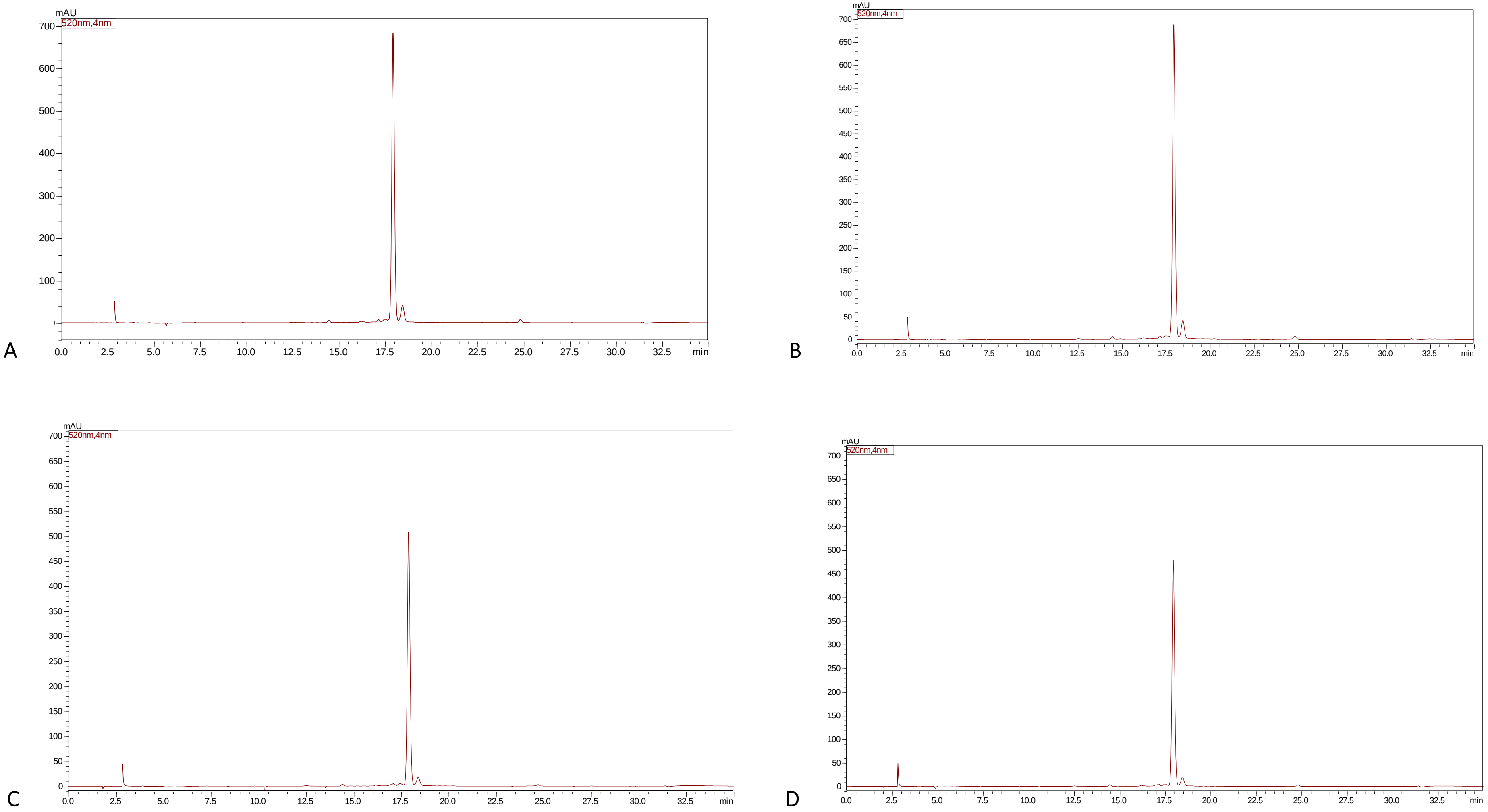

Supplementary figure S1 Chromatograms of Undigested Natal plum juice samples at 520 nm (anthocyanins), A (NMaPeaPsy: Natal plum juice + maltodextrin + pea protein isolate + psyllium mucilage), B (NMaPeaPsyB: Natal plum juice + maltodextrin + pea protein isolate + psyllium mucilage + *Ltp. plantarum 75*), C (NM: Natal plum juice) & D (NMB: Natal plum juice + *Ltp. plantarum 75*) the peak at 17.96 minutes being Cyanidin 3-Sambubioside

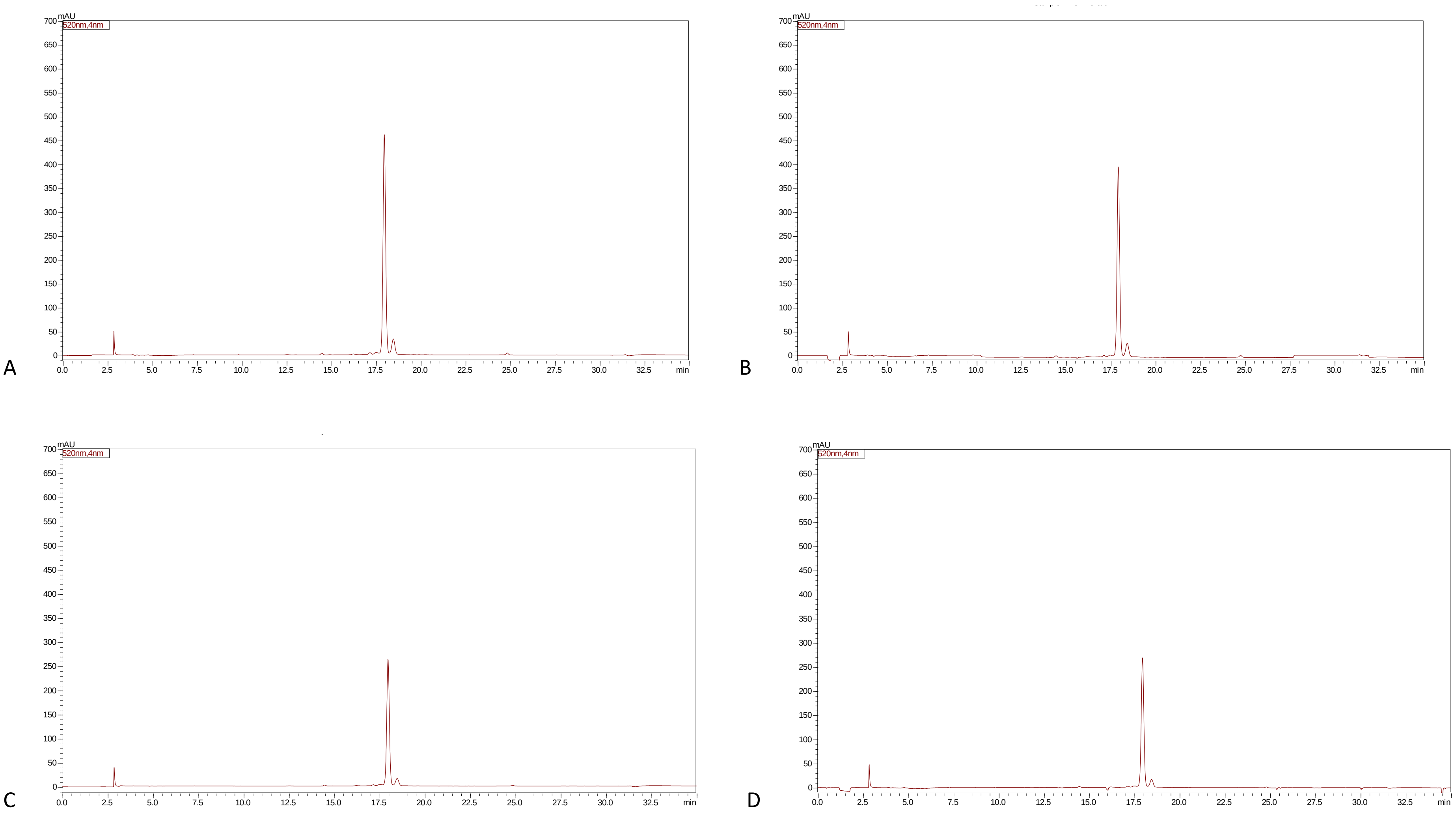

Supplementary figure S2 Chromatograms of Gastric digested Natal plum juice samples at 520 nm (anthocyanins), A (NMaPeaPsy: Natal plum juice + maltodextrin + pea protein isolate + psyllium mucilage), B (NMaPeaPsyB: Natal plum juice + maltodextrin + pea protein isolate + psyllium mucilage + *Ltp. plantarum 75*), C (NM: Natal plum juice) & D (NMB: Natal plum juice + *Ltp. plantarum 75*) the peak at 17.96 minutes being Cyanidin 3-Sambubioside

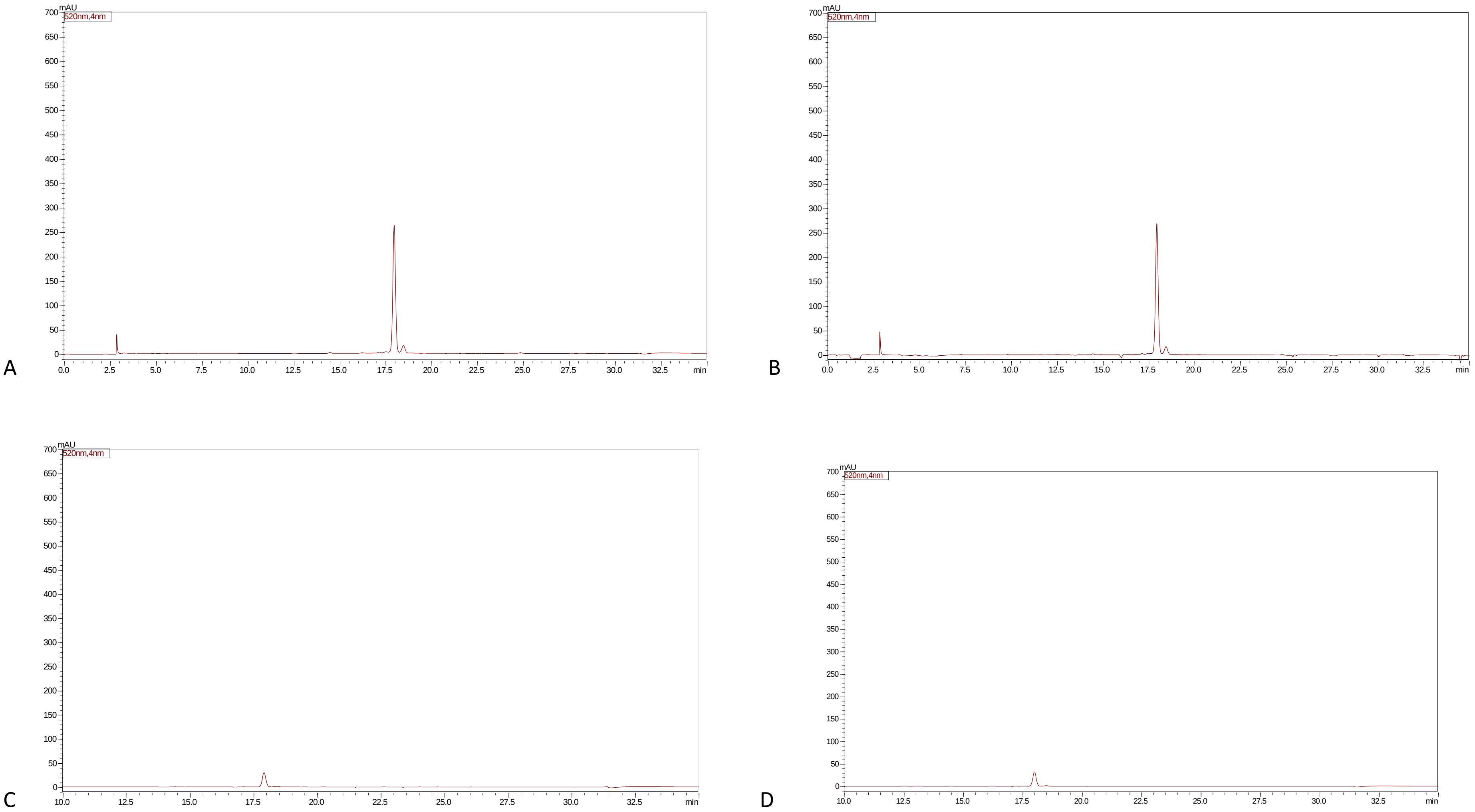

Supplementary figure S3 Chromatograms of Intestinal digested Natal plum juice samples at 520 nm (anthocyanins), A (NMaPeaPsy: Natal plum juice + maltodextrin + pea protein isolate + psyllium mucilage), B (NMaPeaPsyB: Natal plum juice + maltodextrin + pea protein isolate + psyllium mucilage + *Ltp. plantarum 75*), C (NM: Natal plum juice) & D (NMB: Natal plum juice + *Ltp. plantarum 75*) the peak at 17.96 minutes being Cyanidin 3-Sambubioside

NM

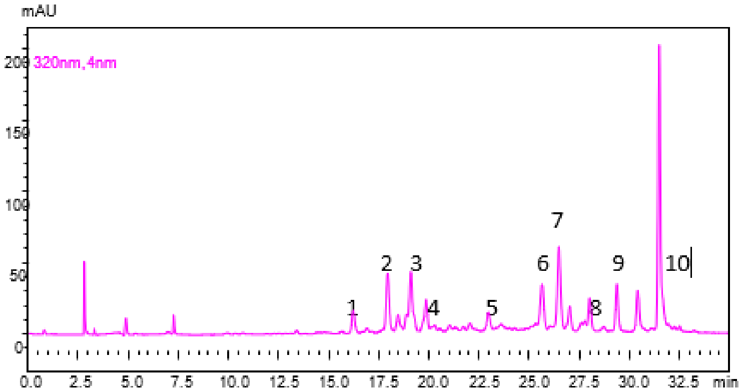

NMB

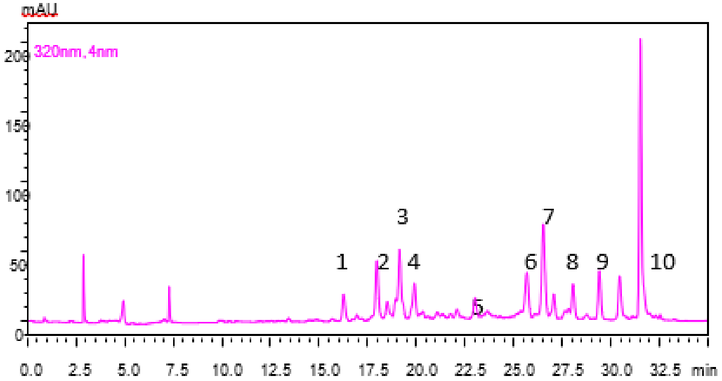

NMaPeaPsy

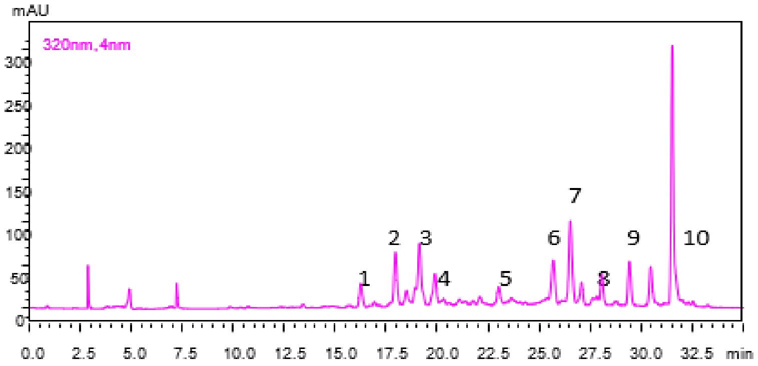

NMaPeaPsyB

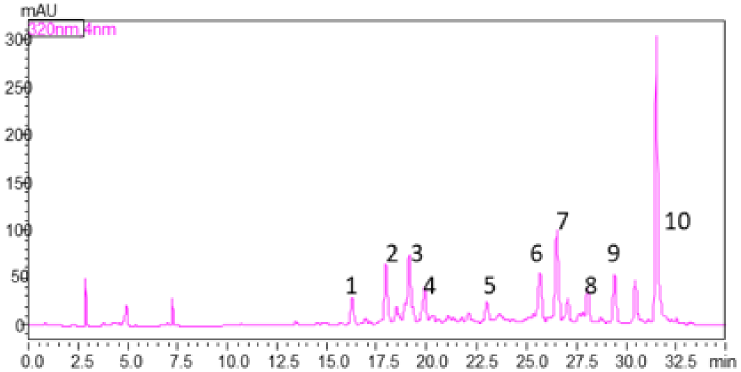

Supplementary figure S4 Chromatograms of the identification of phenolic acids and flavonoids in Natal plum juice samples at 320 nm, NMaPeaPsy: Natal plum juice + maltodextrin + pea protein isolate + psyllium mucilage, NMaPeaPsyB: Natal plum juice + maltodextrin + pea protein isolate + psyllium mucilage

+ *Ltp. plantarum 75*, NM: Natal plum juice & NMB: Natal plum juice + *Ltp. plantarum 75*. 1: protocatechuic acid, 2: (anthocyanin), 3: chlorogenic acid, 4: catechin, 5: caffeic acid, 6: ellagic acid, 7: quercetin 3-glucoside 8: p-Coumaric, 9: ferulic acid & 10: dicaffeoylquinic acid.
